# Radiative Colloidal Investigation for Thermal Transport by Incorporating the Impacts of Nanomaterial and Molecular Diameters (d_Nanoparticles_, d_Fluid_): Applications in Multiple Engineering Systems

**DOI:** 10.3390/molecules25081896

**Published:** 2020-04-20

**Authors:** Naveed Ahmed, Adnan, Umar Khan, Syed Tauseef Mohyud-Din, Yu-Ming Chu, Ilyas Khan, Kottakkaran Sooppy Nisar

**Affiliations:** 1 Department of Mathematics, Faculty of Sciences, HITEC University, Taxila Cantt 47070, Pakistan; nidojan@gmail.com; 2 Department of Mathematics, Mohi-ud-Din Islamic University, Nerian Sharif AJ&K 12080, Pakistan; adnan_abbasi89@yahoo.com; 3 Department of Mathematics and Statistics, Hazara University, Mansehra 21120, Pakistan; umar_jadoon4@yahoo.com; 4 Department of Mathematics, University of Multan, Multan 60000, Pakistan; syedtauseefs@hotmail.com; 5 Department of Mathematics, Huzhou University, Huzhou 313000, China; chuyuming@zjhu.edu.cn; 6 Faculty of Mathematics and Statistics, Ton Duc Thang University, Ho Chi Minh City 72915, Vietnam; 7 Department of Mathematics, College of Arts and Sciences, Prince Sattam bin Abdulaziz University, Wadi Aldawaser 11991, Saudi Arabia; n.sooppy@psau.edu.sa

**Keywords:** thermal enhancement, entropy generation, Al_2_O_3_-H_2_O colloidal suspension, thermal radiations, freezing temperature

## Abstract

Thermal enhancement and irreversible phenomena in colloidal suspension (Al_2_O_3_-H_2_O) is a potential topic of interest from the aspects of industrial, mechanical and thermal engineering; heat exchangers; coolant car radiators; and bio-medical, chemical and civil engineering. In the light of these applications, a colloidal analysis of Al_2_O_3_-H_2_O was made. Therefore, a colloidal model is considered and treated numerically. The significant influences of multiple parameters on thermal enhancement, entropy generation and Bejan parameter are examined. From the presented colloidal model, it is explored that Al_2_O_3_-H_2_O is better for the applications of mechanical and applied thermal engineering. Moreover, fraction factor tiny particles are significant parameters which enhanced the thermal capability of the Al_2_O_3_-H_2_O suspension.

## 1. Introduction

The heat-transfer inspection in nanofluids gained much interest from the researchers, engineers, industrialist and scientists. Nanofluids have potential heat-transfer characteristics that make them superior to regular liquids, and researchers, engineers and scientists prefer the applications of nanofluids for industrial and technological zones. When the regular liquids are used in the aforementioned areas, these fluids fail to accomplish the production processes for many industrial items which need large heat transfer. Therefore, in such a scenario, the nanofluids are reliable fluids to use that provide the required heat to accomplish the production of different items in the industries. Nanofluids are a potential field of interest, and the researchers focused their attention on the uses of these rich heat-transfer fluids in different areas and in daily life, as well. These fluids are positively used in coolant car radiators, medical sciences, drugs, electronics, the production of computer chips, civil engineering, biotechnology, electrical engineering and in many other industries. 

It is a fact that the heat transportation in the conventional liquids can be enhanced by adding the tiny (nano-sized) particles of various metals (Cu, Ag), carbon nanotubes (multi-wall carbon nanotubes (MWCNTs) and single-wall carbon nanotubes (SWCNTs)), ferromagnetic (Fe_3_O_4_) and oxides of different metals (γAl_2_O_3_, CuO, Al_2_O_3_) in the conventional, such that aforementioned nanoparticles and conventional liquid are thermally in equilibrium. The reason for the high heat-transfer characteristics of nanofluids is the thermal conductivity that made these fluids superior from regular liquids (water, engine oil, ethylene glycol EG, kerosene oil, etc.). The large thermal conductivity of the aforementioned metals and their oxides enhances the thermal conductivity of the resultant mixture that is called nanofluid. Therefore, the nanofluids comprising the high thermal-conductivity characteristics have high heat-transfer characteristics and are better for industrial and technological purposes. 

Recently, Bhatti et al. [1] reported the influences of multiple parameters in the flow regimes for radiative-ferromagnetic-material-based nanofluid. The study carried out for porous stretchable sheet with cross diffusion gradients. The study of radiative Jeffery nanofluid by incorporating the effects of chemical reaction and cross diffusion effects in the existence of ohmic heating for non-Newtonian fluid was examined in [2,3], respectively. The role of numerous heat-transport mechanisms on thermal conductivity of the colloidal fluids is presented in [4]. The study of several flow parameters on the behavior of entropy generation for carbon nanotubes (CNTs) nanofluid between rotating disks is discussed in [5].

By considering the thermal conductivity as an important ingredient in the nanofluids’ heat-transfer phenomena, scientists and researchers focused to develop the theoretical correlations for nanofluids and suggested many thermal conductivity models, by incorporating different factors like temperature, diameter of the nanoparticles, molecular diameter and volume fraction factor of the tiny particles, etc. A thermal conductivity model for different shapes of the nanoparticles (platelets, cylinders, spherical, blades and bricks) was developed by Hamilton [6]. The parameter n is adjusted in the model that leads to different shapes for distinct values. A particular thermal conductivity correlation is achieved by using the temperature influences developed by Kleinstreuer and Koo [7,8]. Bruggemann [9] proposed a thermal conductivity relation particularly for spherical type tiny particles at high volume fraction factor. Li and Peterson [10] developed particular thermal conductivity relation which works for the composition H_2_O/Al_2_O_3_. To enhance the effectiveness of the model, they incorporated the fraction factor and temperature influences. 

Patel et al. [11] proposed reliable thermal conductivity model based on the tiny particles’ diameter, only for oxides and metallic tiny particles. For the thermal improvement in the mixture of H_2_O/Al_2_O_3_, the model is reported in [12]. The freezing temperature factor is incorporated in the respective thermal conductivity relation and found fascinating alterations in the heat transfer. Thermal conductivity model for water composed by gold nanoparticles (Ag) reported in [13]. The researchers, engineers and scientists inspired by the potential heat-transfer characteristics in a newly developed class of fluids (nanofluids) and focused on the applications of these fluids for different purposes. 

The flow nanofluid by using water as host liquid between parallel disks is reported in [14]. For fascinating results for the nanofluid velocity and temperature, the influences of unsteady factor and slip effects are incorporated in the governing model. They handled the model analytically and detected the results for the flow regimes by fluctuating the emerging flow parameters. The solutal flow of nanofluid squeezed between two parallel disks in porous media is examined in [15]. They considered the phenomena of imposed magnetic field, momentum slip and thermal jump flow conditions in the model and detected the improvement in the nanofluid heat transfer characteristics. The influences of momentum slip on the nanofluid velocity between parallel disks is examined in [16]. They adopted numerical way for the problem treatment and reported the results for the flow regimes and heat-transfer rate, as well. The mixed convection flow of unsteady nature between parallel disks is presented in [17]. They explored the results for nanofluid characteristics, like velocity, temperature and heat-transfer characteristics. 

Ahmed et al. [18] detected the velocity, temperature and heat transfer-behavior in nanofluids squeezed between parallel disks. They reported the results for different nanoparticle-based nanofluids and found an improved heat-transfer rate. Azimi and Riazi [19] investigated the heat-transfer behavior in GO/H_2_O nanofluid between parallel disks. For mathematical treatment of the nanofluid model, they adopted a Galerkin-based homotopy analysis method and reported the results for the flow characteristics. The analysis of variable fluid characteristics in flow squeezed between parallel disks by incorporating the influences of magnetic field was detected in [20]. Ahmed et al. [21] detected the intensified heat transfer behavior in γ-nanofluids between parallel rotating plates and presented fascinating behavior of the nanofluids velocity and temperature, by varying the emerging flow quantities in the model.

The effective thermal conductivity is a key ingredient for the improvement of thermal transport in the analysis of nanofluids. Therefore, numerous thermal conductivity correlations can be achieved by incorporating different factors, like tiny particles geometries, temperature factor, molecular diameter, tiny particles diameter and freezing temperature [6,7,8,22]. The tiny particles diluted in the host liquid enhances the surface area of thermal transport, effective heat capacity of the host liquid and thermal conductivity. Therefore, thermal transportation in the nanofluids is enhanced.

The entropy generation is one of the rich topics in the field of fluid dynamics. The amount of entropy due to irreversible processes is termed as entropy production. These are thermal and mass transport processes, fluid flow, movement of the bodies, mixture of nanomaterials and liquids, and heat exchange and in thermal mechanics, like air conditioners, power plants, refrigerators, heat engines, etc. On the basis of entropy production, another significant physical-quantity defined termed as Bejan number in the scientific domain of fluid dynamics. The Bejan physical describes the ratio between thermal transport irreversibility to the total irreversibility produced due to the thermal transport and friction between the fluid particles. Therefore, entropy production and the Bejan number are extensively used in the field of fluid dynamics and aforementioned industrial applications. The coupling of entropy production and Bejan effects with numerous nanofluid models is very significant, and many researchers and engineers focus on the analysis of such nanofluid models.

From the literature study, we detected that the thermal enhancement and the behavior of Bejan effects, by considering the thermal radiations and the diameters of nanoparticles and molecules in dissipative nanofluid between parallel disks, have been not examined so far. Therefore, this study was made to conduct this important research.

## 2. Results and Discussion

### 2.1. ${Al}_{2}O_{3}-H_{2}O$ Axial and Radial Velocity Behavior

The behavior of flow characteristics significantly alters for multiple parameters embedded in the flow model Al_2_O_3_-H_2_O colloidal mixture. These are stretching parameters (A_1_, A_2_), rotational parameter Ω, Reynolds number (Re), Prandtl number (Pr) and radiative parameter (Rd). Therefore, the behavior of the velocities (axial (F), radial (F’) and tangential velocity (G)), thermal transport β(η), shear stresses, entropy generation and Bejan effects for Al_2_O_3_-H_2_O colloidal mixture are painted. The impacts of fraction factor of the tiny particles of Al_2_O_3_ are discussed, and we found fascinating alterations in the effective characteristics like density, heat capacitance and thermal conductivity of the nanofluid.

**Figure 1:**
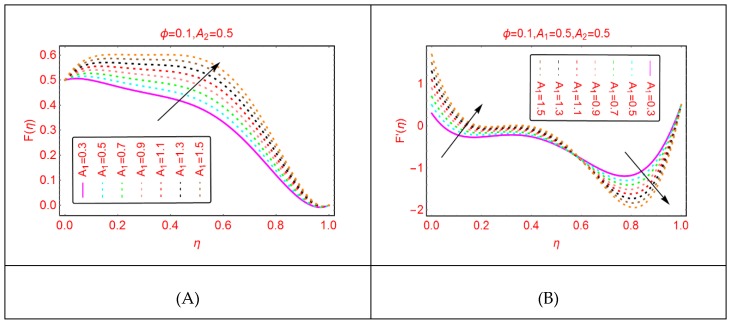
The velocity distribution for $A_{1}$. (**A**): F(η); (**B**): $F^{\prime }\left(\eta \right)$.

The influences of stretching parameter A_1_ on the axial (F) and radial velocities (F’) of Al_2_O_3_-H_2_O colloidal mixture are painted in Figure 1. It is noticed that the velocity, F, due to the stretching of the lower disk, enhances abruptly in the locality of η=0. Physically, when the lower disk is stretched, the fluid particles of Al_2_O_3_-H_2_O adjacent to the disk surface drag and stretching of the disk provides the extra momentum to the molecules consequently the momentum rises, leading to abrupt changes in the velocity, F. The maximal increment in the velocity is observed in the region 0.0≤η≤0.6. In the rest of the portion, these alterations become slow because small disturbance in the fluid molecules away from the lower disk occurred. Therefore, the rise in the velocity becomes slow.

**Figure 2:**
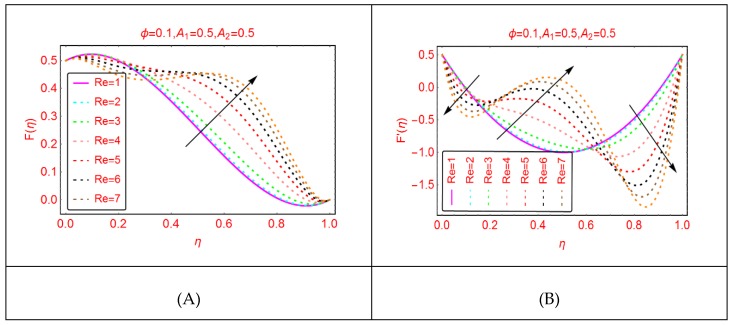
The velocity distribution for Re. (**A**): F(η); (**B**): $F^{\prime }\left(\eta \right)$.

**Figure 3:**
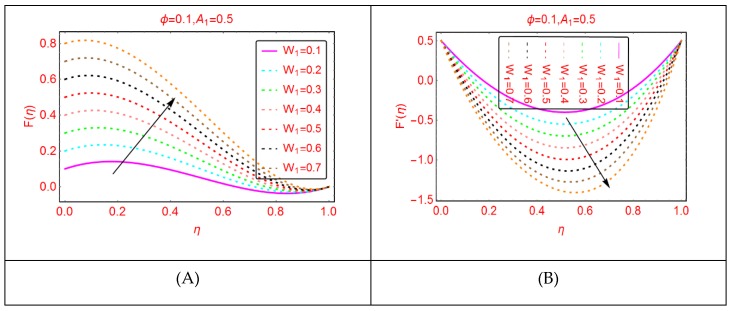
The velocity distribution for $w_{1}$. (**A**): F(η); (**B**): $F^{\prime }\left(\eta \right)$.

The Reynolds number, which is the relationship between the inertial and viscous forces, is a significant flow parameter in the study of fluid dynamics. Figure 2 paints the behavior of Al_2_O_3_-H_2_O velocity for multiple values of Reynolds number. These effects are painted in Figure 2. It is detected that, by increasing the Reynolds number (Re), the velocity enhances. Physically, it can be justified by the fact that, for more viscous fluid, the Reynolds number upturns, and consequently the velocity abruptly increases. The radial velocity, F’, shows dual behavior for multiple Reynolds numbers in the region of interest. Figure 3 paints the velocities’ behavior for multiple w_1_ parameters. It is detected that the axial and radial velocity showed reverse trends. The axial velocity, F, near the lower end enhances quickly and exhibits almost minimal behavior, if examined near the upper end. The decreasing trend in the radial velocity of Al_2_O_3_-H_2_O nanofluid is observed. In the vicinity of the middle portion of the disks, maximal increasing trends are observed.

**Figure 4:**
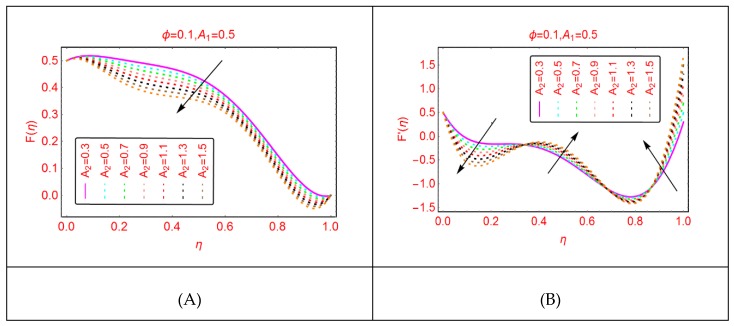
The velocity distribution for $A_{2}$. (**A**): F(η); (**B**): $F^{\prime }\left(\eta \right)$.

Figure 4 paints the alterations in the velocities for the stretching of the upper disk. Due to stretching of the upper disk, the velocities decline, and minimal decrement is observed near the upper disk. In rest of the portion, these trends are abrupt. Physically, due to the stretching of the upper disk, the fluid molecules adjacent to the disk are disturbed, leading to upturns in the momentum. Therefore, the velocities drop slowly in comparison with the rest of the portion.

### 2.2. ${Al}_{2}O_{3}-H_{2}O$ Tangential Velocity Behavior

This subsection comprises the alterations in the tangential velocity G(η) for Al_2_O_3_-H_2_O nanofluid for stretching parameters and the Reynolds number.

**Figure 5:**
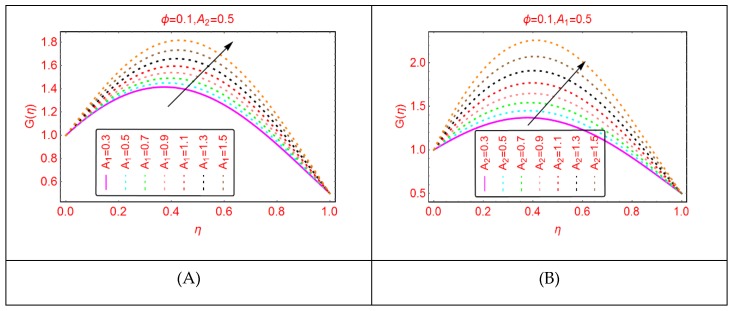
The velocity distribution G(η). (**A**): $A_{1}$; (**B**): $A_{2}$.

**Figure 6:**
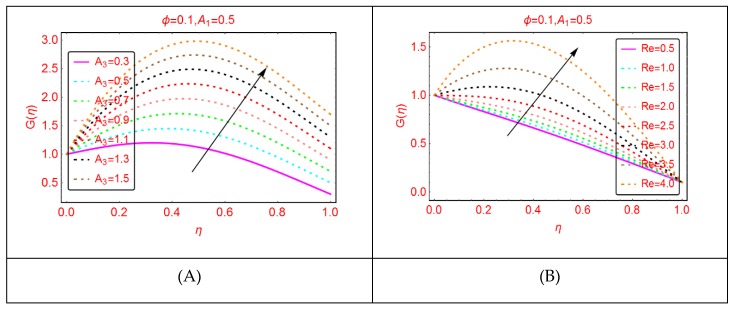
The velocity distribution G(η). (**A**): $A_{3}$; (**B**): Re.

The influences of stretching properties (A_1_ and A_2_) of the upper and lower disks are painted in Figure 5. It is surveyed that the tangential velocity rises quickly due to stretching of the disks. Physically, it can be justified that the Al_2_O_3_-H_2_O nanofluid and the disks rotate together and in the same time the disks stretched. Due to stretching and rotation of the disks, the movement of the fluid particles enhance, and the momentum of the Al_2_O_3_-H_2_O nanofluid increases, and as a result, the tangential velocity also increases. Near the lower and upper end, these alterations are quite slow; these are due to the frictional force between the fluid particles and the disks surface. Similarly, Figure 6 paints the tangential velocity, G(η), behavior for A_3_ and the Reynolds number. It is inspected that G(η) upturns for both A_3_ and Re. The quick alterations in the tangential velocity of Al_2_O_3_-H_2_O nanofluid are examined for A_3_ near the upper end.

### 2.3. ${Al}_{2}O_{3}-H_{2}O$ Thermal Behavior

This subsection is related to the analysis of thermal behavior of Al_2_O_3_-H_2_O nanofluid by altering Br, Ec and Re, respectively.

**Figure 7:**
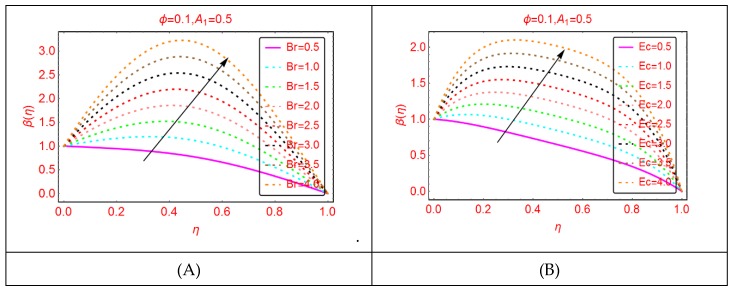
The thermal distribution β(η). (**A**): Br; (**B**): Ec.

**Figure 8:**
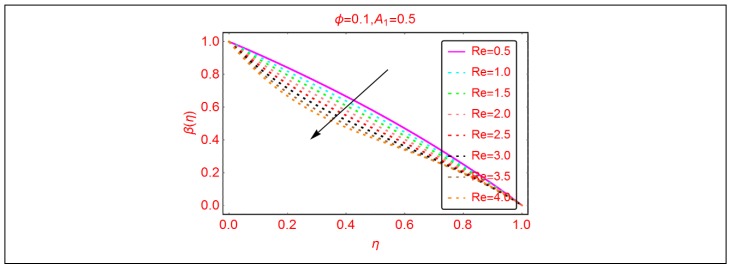
The thermal distribution β(η) for Re.

The nanofluids have potential heat-transportation capability in comparison with regular liquid. Therefore, these fluids are better for industrial and technological purposes. The behavior of thermal transport β(η) against Br and the Eckert number is painted in Figure 7. The phenomenon of viscous dissipation is of significant interest in the study of fluid dynamics. From Figure 8, it is pointed that the temperature β(η) upturns for more dissipative nanofluid. Physically, for more dissipative Al_2_O_3_-H_2_O, nanofluid thermal energy enhances, leading to increment in the temperature β(η). Similarly, increasing trends for β(η) are pointed by altering Br number. Figure 8 reveals that, for the Reynolds number, the temperature β(η) declines. The small decrement is pointed at low Reynolds values, whereas it is rapid for higher ones.

### 2.4. Bejan Effects and Entropy Generation

**Figure 9:**
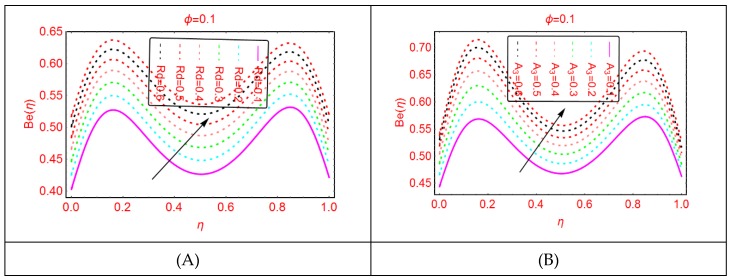
The distribution of Be(η). (**A**): Rd; (**B**): $A_{3}$.

**Figure 10:**
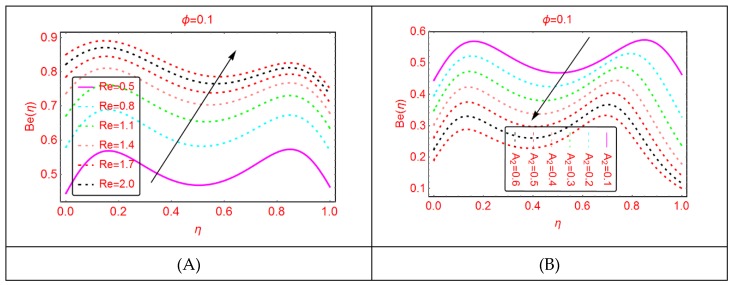
The distribution of Be(η). (**A**): Re; (**B**): $A_{2}$.

**Figure 11:**
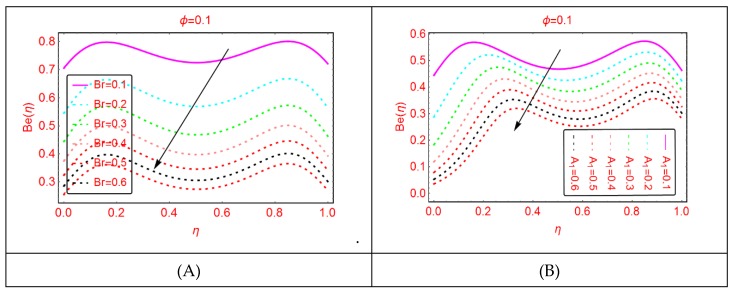
The distribution of Be(η). (**A**): Br; (**B**): $A_{1}$.

**Figure 12:**
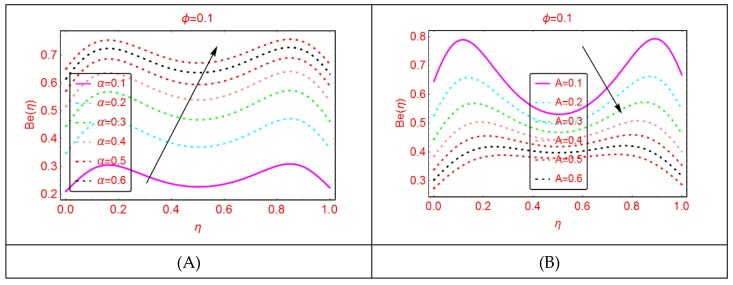
The distribution of Be(η). (**A**): α; (**B**): A.

**Figure 13:**
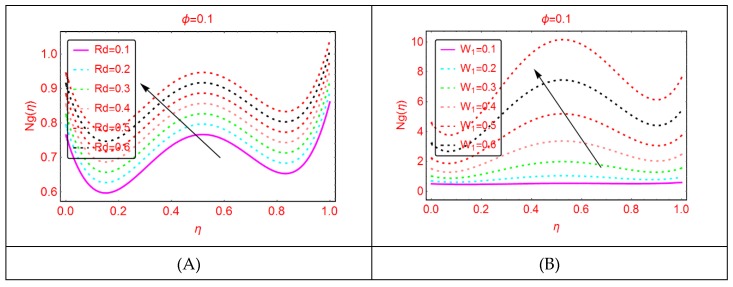
The distribution of $N_{g}\left(\eta \right)$. (**A**): Rd; (**B**): $w_{1}$.

**Figure 14:**
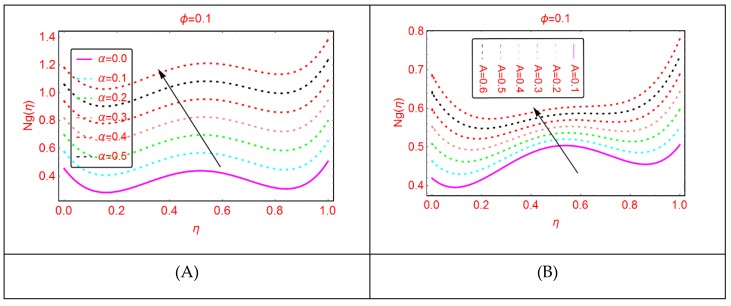
The distribution of $N_{g}\left(\eta \right)$. (**A**): α; (**B**): A.

**Figure 15:**
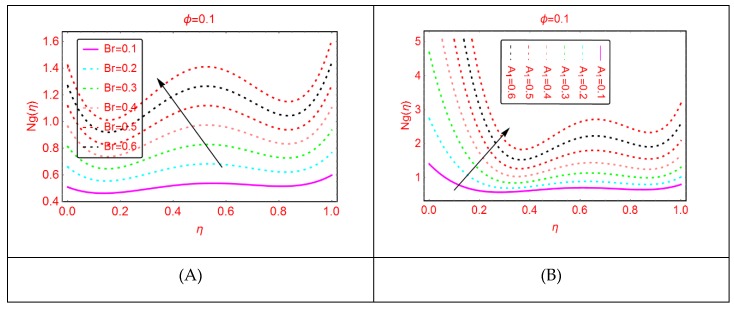
The distribution of $N_{g}\left(\eta \right)$. (**A**): *Br*; (**B**): *A*_1_.

**Figure 16:**
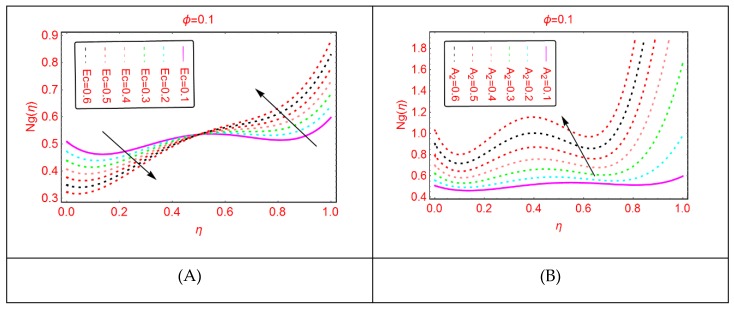
The distribution of $N_{g}\left(\eta \right)$. (**A**): Ec; (**B**): $A_{2}$.

**Figure 17:**
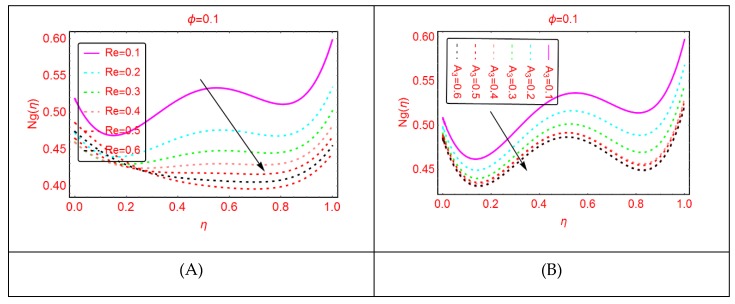
The distribution of $N_{g}\left(\eta \right)$. (**A**): Re; (**B**): $A_{3}$.

Entropy and the phenomena of Bejan effects are very significant in the research area of fluid mechanics. This phenomenon has a wide class of uses in moving bodies, thermal machines, heat exchange, thermal characteristics in nanofluids and irreversible thermodynamics. The entropy production is described as the entropy during irreversible processes. The influences of multiple parameters related to entropy production and Bejan effects are elaborated in Figure 9, Figure 10, Figure 11, Figure 12, Figure 13, Figure 14, Figure 15, Figure 16 and Figure 17, for multiple parameters over the region of interest.

It is explored that the Bejan parameter enhances for growing A_3_ and Re abruptly in the middle of the disks, where for the more radiative colloidal mixture Al_2_O_3_-H_2_O, prominent alterations in the Bejan parameter are examined near both ends. These influences are elaborated in Figure 9 and Figure 10. From the analysis, it is also explored that, for A_2_, B_r_, A_1_ and A, the Bejan effects drop between the disks.

The entropy production against imposed radiation parameter is depicted in Figure 13. It is detected that, for more radiative mixture of Al_2_O_3_-H_2_O, the entropy N_g_(η) enhances abruptly. Physically, it means that, due to more radiative Al_2_O_3_-H_2_O, internal energy source enhances, allowing the large momentum to the Al_2_O_3_-H_2_O molecules and therefore diffusion phenomena among the tiny particles upturns and N_g_(η) increases. Similarly, for w_s_, α, A and B_r_, the entropy enhances. For stretching quantity A_1_, the abrupt increasing pattern of N_g_(η) is examined. The physical reason behind this is the stretching of the lower disk. Due to stretching of the lower end, the Al_2_O_3_-H_2_O molecules are disturbed, allowing for the abrupt increment in the N_g_(η). For more dissipative Al_2_O_3_-H_2_O mixture, dual pattern of N_g_(η) is detected. Near the lower end N_g_(η) declines for stronger dissipative parameter and also for Re and A_3_, N_g_(η) declines quickly.

### 2.5. Thermophysical Characteristics

**Figure 18:**
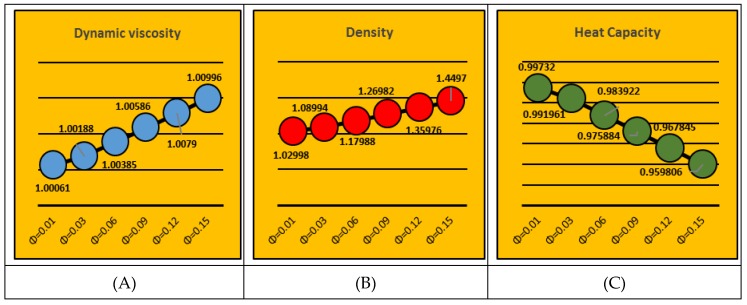
Thermophysical properties behavior for ϕ. (**A**):$\;\mu_{nf}$; (**B**): $\rho_{nf}$; (**C**): ${\left(\rho c_{p}\right)}_{nf}$.

In the colloidal studies, the fraction factor, ϕ, is a significant ingredient which alters the thermal transportation in the nanofluid. The changes in the dynamic viscosity (μ_nf_), density (ρ_nf_) and heat capacity (ρc_p_)_nf_ of nanofluid play an important role in the flow regimes. Therefore, Figure 18 is painted to analyze the behavior of aforementioned quantities by altering the fraction factor, ϕ. For the study under consideration, the feasible range of the fraction factor is 0.0<ϕ≤0.2. It is pointed that the dynamic viscosity and density upturns for ϕ and drops in (ρc_p_)_nf_ are pointed for multiple values of ϕ.

### 2.6. Engineering Quantities

**Figure 19:**
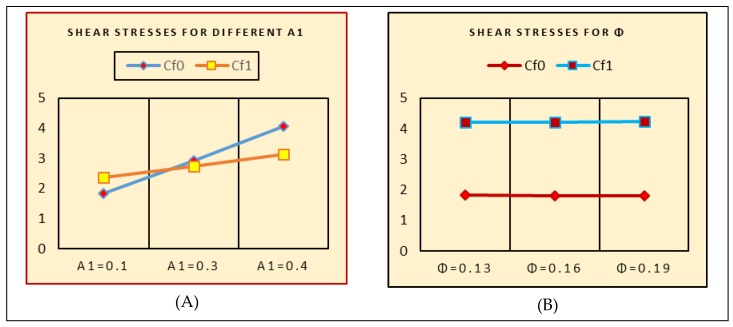

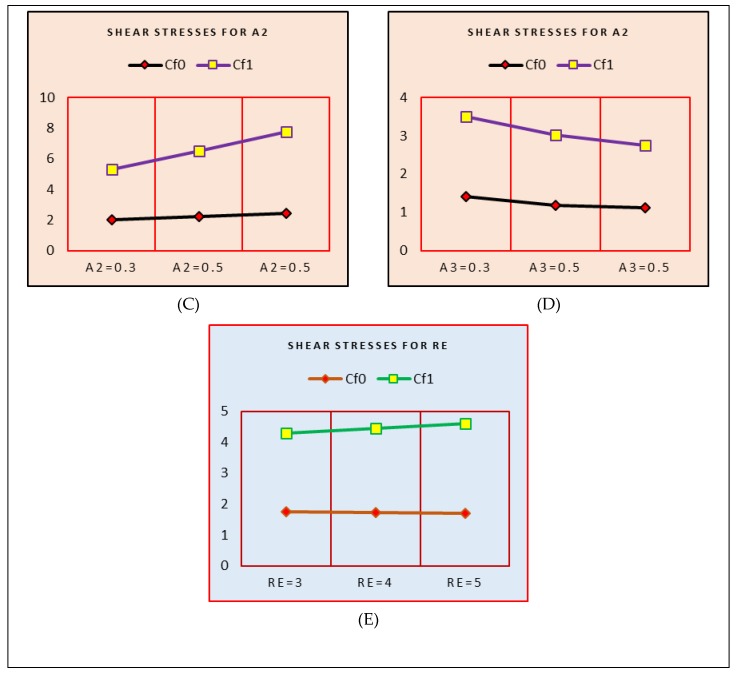
Shear stresses for. (**A**): $A_{1}$; (**B**): ϕ; (**C**): $A_{2}$; (**D**): $A_{3}$; (**E**): Re.

The surface shear stresses due to varying flow quantities are important from engineering point of view. The behavior of surface shear stresses due to stretching of the lower and upper disks (A_1_ and A_2_) and fraction factor ϕ are elaborated in Figure 19. For the stretching of the disks, larger trends of the shear stresses at the surface are pointed. Physically, it means that the stretching of the disks allows for more fluid particles to be at the surface; therefore, the shear stresses increases. For the fraction factor, slow trends are observed for shear stresses at the disk’s surface. For upper stretchable disk (A_2_), the surface stresses rapidly decline at the lower end, while these trends are slow at the upper disk because the upper disk being stretched allows the fluid particles at the disk surface. A significant transport of the shear stresses is pointed at the upper disk for Re. Table 1 elaborates on the numerical computations for the shear stresses for multiple parameters.

## 3. Materials and Methods

### 3.1. Statement and Geometry of the Model

**Figure 20:**
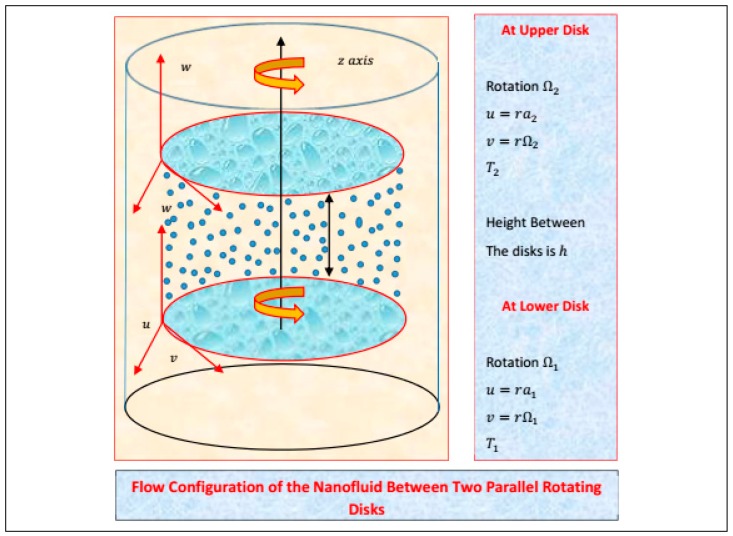
The flow configuration of Al_2_O_3_-H_2_O nanofluid between parallel rotating disks.

The flow of nanofluid by contemplating the influences of nanoparticles and molecular diameters is taken between parallel rotating disks. It is understood that host liquid (water) and nanoparticles Al_2_O_3_ are thermally compatible. The disks are separated by a height, h. The lower and upper disks are rotating with velocities Ω_1_ and Ω_2_, respectively. Moreover, u = ra_1,_ vΩ_1_, u = ra_2_ and v = rΩ_2_ are the velocities of lower and upper disks, respectively. Additionally, the flow configuration of the nanofluid model under consideration is portrayed in Figure 20.

### 3.2. Governing Colloidal Model

The colloidal flow of nanofluid by considering the nanomaterial and molecular diameters is taken between two parallel rotating disks. The following assumptions are made for the model under consideration:

- The flow is viscous and incompressible;
- H_2_O is taken as host liquid;
- The nanomaterial of Al_2_O_3_ is taken;
- The host liquid and nanomaterial are thermally in equilibrium;
- There is no slip condition;
- Thermal radiations are imposed on the nanofluid flow.

On the basis of above highlighted assumptions, the dimensional model which governs the nanofluid flow between parallel disks takes the following form [5]:

(1) $$\frac{\partial u^{*}}{\partial r}+\frac{\partial w^{*}}{\partial z}+\frac{u^{*}}{r}=0,$$

(2) $$u^{*}\frac{\partial u^{*}}{\partial r}+w^{*}\frac{\partial u^{*}}{\partial z}-\frac{v^{*}}{r^{2}}+\frac{1}{\rho_{nf}^{*}}\frac{\partial p^{*}}{\partial r}-\nu_{nf}^{*}\left(\frac{\partial^{2}u^{*}}{\partial r^{2}}+\frac{1}{r}\frac{\partial u^{*}}{\partial r}+\frac{\partial^{2}u^{*}}{\partial z^{2}}-\frac{u^{*}}{r^{2}}\right)=0,$$

(3) $$u^{*}\frac{\partial v^{*}}{\partial r}+w^{*}\frac{\partial v^{*}}{\partial z}+\frac{v^{*}u^{*}}{r}-\nu_{nf}^{*}\left(\frac{\partial^{2}v^{*}}{\partial r^{2}}+\frac{1}{r}\frac{\partial v^{*}}{\partial r}+\frac{\partial^{2}v^{*}}{\partial z^{2}}-\frac{v^{*}}{r^{2}}\right)=0,$$

(4) $$u^{*}\frac{\partial w^{*}}{\partial r}+w^{*}\frac{\partial w^{*}}{\partial z}+\frac{1}{\rho_{nf}^{*}}\frac{\partial p^{*}}{\partial z}-\nu_{nf}^{*}\left(\frac{\partial^{2}w^{*}}{\partial r^{2}}+\frac{\partial^{2}w^{*}}{\partial z^{2}}+\frac{1}{r}\frac{\partial w^{*}}{\partial r}\right)=0,$$

(5) $${\left(\rho c_{p}\right)}_{nf}\left(u^{*}\frac{\partial T^{*}}{\partial r}+w^{*}\frac{\partial T^{*}}{\partial z}\right)-\left(k_{nf}^{*}+\frac{16\sigma^{*}T_{\infty }^{3}}{3k^{*}}\right)\left(\frac{\partial^{2}T^{*}}{\partial r^{2}}+\frac{1}{r}\frac{\partial T^{*}}{\partial r}+\frac{\partial^{2}T^{*}}{\partial z^{2}}\right)-\mu_{nf}^{*}\Phi^{*}=0$$

where Φ* shows the dissipative effects and is described by the following formula:

(6) $$\Phi^{*}=2\left({\left(\frac{\partial u^{*}}{\partial z}\right)}^{2}+{\left(\frac{u^{*}}{r}\right)}^{2}+{\left(\frac{\partial w^{*}}{\partial z}\right)}^{2}\right)+{\left(\frac{\partial v^{*}}{\partial z}\right)}^{2}+{\left(\frac{\partial w^{*}}{\partial r}+\frac{\partial u^{*}}{\partial z}\right)}^{2}+{\left(r\frac{\partial }{\partial r}\left(\frac{v^{*}}{r}\right)\right)}^{2}$$

The flow conditions at the boundaries of the disks (lower and upper) are as follows.

For nondimensionalization of model, it is significant to define the invertible transformations. Therefore, the following transformations are defined that support the model:

(7) $$\begin{array}{c} \mathrm{At}\;\;z=0\\ u^{*}=ra_{1}\\ v^{*}=r\Omega_{1}\\ w^{*}=w_{0}^{*}\\ T^{*}=T_{1}\\ at\;z=h\\ u^{*}=ra_{2}\\ v^{*}=r\Omega_{2}\\ w^{*}=0\\ T^{*}=T_{2} \end{array}\},$$

(8) $$\begin{array}{c} u^{*}=r\Omega_{1}F^{\prime }\left(\frac{z}{h}\right)\\ v^{*}=r\Omega_{1}G\left(\frac{z}{h}\right)\\ w^{*}=-2h\Omega_{1}F\left(\frac{z}{h}\right)\\ \beta \left(\frac{z}{h}\right)=(T^{*}-T_{2})/\left(T_{1}-T_{2}\right)\\ p^{*}=\rho_{f}\nu_{f}\Omega_{1}\left(P\left(\frac{z}{h}\right)+0.5{\left(\frac{r}{h}\right)}^{2}\epsilon \right)\\ \eta =z/h \end{array}\},$$

where, u*, v* and w* are the velocity components; $\Omega_{1}$ and $\Omega_{2}$ present the rotation of the disks; *h* is the height between the disks; *T*_1_ and *T*_2_ are the temperature at the lower and upper end, respectively; β indicates the dimensionless temperature; and η is an invertible variable.

### 3.3. Effective Characteristics

The nanofluids are reliable for better thermal transport due to their effective characteristics, such as effective density, heat capacity, dynamic viscosity and thermal conductivity. Therefore, the following models are incorporated [22]:

(9) $$\rho_{nf}^{*}=\left[\left(1-\phi \right)+\frac{\phi \rho_{p}}{\rho_{f}}\right]\rho_{f},\;$$

(10) $${\left(\rho C_{p}\right)}_{nf}=\left[\left(1-\phi \right)+\frac{\phi {\left(\rho C_{p}\right)}_{p}}{{\left(\rho C_{p}\right)}_{f}}\right]{\left(\rho C_{p}\right)}_{f},$$

(11) $$\mu_{nf}^{*}=\mu_{f}{\left(1-34.87{\left(\frac{d_{particle}}{d_{fluid}}\right)}^{-0.3}\phi^{1.03}\right)}^{-1}.$$

(12) $$k_{nf}^{*}=k_{f}\left(1+4.4Re_{b}^{0.4}\overset{0.66}{\mathrm{Pr}}{\left(\frac{T}{T_{freezing}}\right)}^{10}{\left(\frac{k_{p}}{k_{f}}\right)}^{0.03}\phi^{0.66}\right),$$

where *nf* stands for nanofluid; and *k_f_* and *k_p_* are the thermal conductivities of the host liquid and tiny particles, respectively. Further, $\rho_{nf}^{*}$, ${\left(\rho C_{p}\right)}_{nf}$, $\mu_{nf}^{*}$ and $k_{nf}^{*}$ are effective density, heat capacity, dynamic viscosity and thermal conductance of the nanofluid. The fraction factor of the tiny particles, as denoted by ϕ and 0<ϕ≤0.2, is a feasible range for the study under consideration. In Equation (12), the expression for the Reynolds number (*Re_b_*) appearing due to that effects of Brownian motion is described by the following formula:

(13) $$Re_{b}\left(\mu_{f}\right)=d_{p}\rho_{f}u_{b}^{*},\;$$

Furthermore, u_b_* stands for the velocity of Brownian motion in Equation (13) and is expressed by the following mathematical relation:

(14) $$u_{b}^{*}=2Tk_{b}\left(\pi d_{p}^{2}\mu_{f}\right),\;$$

The Stefan Boltzmann coefficient in Equation (14) is represented by *k_b_*, and its particular value is equal to $1.380648\times 10^{-23}\;\left(JK^{-1}\right)$. Further, the diameter of fluid molecules is given in the form of d_f_ and mathematically expressed in the following way [23]:

(15) $$d_{f}=6M^{*}{\left(N^{*}\rho_{f}\pi \right)}^{-1},\;$$

In Equation (15), M* and N* stand for the molecular weight and Avogadro number, respectively. Further, *d_f_* is computed in the following manner:

(16) $$d_{f}={\left(\frac{6\times 0.01801528}{998.62\times \left(6.022\times 10^{23}\right)\times \pi }\right)}^{\frac{1}{3}}=3.85\times 10^{-10}m,$$

Now, thermophysical characteristics of aforementioned tiny particles and host liquid by comprising the influences of nanoparticles and molecular diameters are described as Table 2 [22]:

### 3.4. Nondimensional Nanofluid Model

The nondimensional analysis of the particular nanofluid model is carried out by means of feasible defined invertible transformations. By plugging the similarity transformations embedded in Equation (8) and required partial derivatives in Equations (1)–(5), the following dimensionless nanofluid model comprising the effects of nanoparticles and molecular diameter is attained:

(17) $$\frac{F^{⁗}}{\left(1-\phi +\frac{\phi \rho_{s}}{\rho_{f}}\right)\left(1-34.87{\left(\frac{d_{particle}}{d_{fluid}}\right)}^{-0.3}\phi^{1.03}\right)}+Re\left(2F^{\prime \prime }F+2G^{\prime }G\right)=0,\;$$

(18) $$\frac{G^{\prime \prime }}{\left(1-\phi +\frac{\phi \rho_{s}}{\rho_{f}}\right)\left(1-34.87{\left(\frac{d_{particle}}{d_{fluid}}\right)}^{-0.3}\phi^{1.03}\right)}+Re\left(2G^{\prime }F-2GF^{\prime }\right)=0,$$

(19) $$\frac{\left(\left(1+4.4Re_{b}^{0.4}\overset{0.66}{\mathrm{Pr}}{\left(\frac{T}{T_{freezing}}\right)}^{10}{\left(\frac{k_{p}}{k_{f}}\right)}^{0.03}\phi^{0.66}\right)+Rd\right)\beta^{\prime \prime }}{Pr}+\frac{2Re}{{\left(\left(1-\phi \right)+\frac{\phi {\left(\rho c_{p}\right)}_{s}}{{\left(\rho c_{p}\right)}_{f}}\right)}^{-1}}\beta^{\prime }F+\;\frac{12BrF^{\prime 2}}{\mathrm{Pr}\left(1-34.87{\left(\frac{d_{particle}}{d_{fluid}}\right)}^{-0.3}\phi^{1.03}\right)}+\frac{Ec\left(G^{\prime 2}+F^{\prime \prime 2}\right)}{\left(1-34.87{\left(\frac{d_{particle}}{d_{fluid}}\right)}^{-0.3}\phi^{1.03}\right)}\;=0.$$

By means of invertible transformations, the dimensional conditions at the lower and upper disks reduced in the following form:

(20) $$\begin{array}{c} at\;lower\;Disk\;\left(\eta =0\right)\\ F\left(\eta \right)=W_{s}\\ F^{\prime }\left(\eta \right)=A_{1}\\ G\left(\eta \right)=1\\ \beta \left(\eta \right)=1\\ P\left(\eta \right)=0\\ at\;upper\;Disk\;\left(\eta =1\right)\\ F\left(\eta \right)=0\\ F^{\prime }\left(\eta \right)=A_{2}\\ G\left(\eta \right)=\Omega \\ \beta \left(\eta \right)=0 \end{array}\},\;$$

Further, the expression for pressure is calculated as follows.

The self-similar parameters appeared in the dimensionless model are described by the following mathematical formulas:

(21) $$P\left(\eta \right)=\frac{\left(-2Re\left(F^{2}-W_{s}^{2}\right)+\frac{\left(F^{\prime }-F^{\prime }\left(0\right)\right)}{\left(1-34.87{\left(\frac{d_{particle}}{d_{fluid}}\right)}^{-0.3}\phi^{1.03}\right)\left(1-\phi +\frac{\phi \rho_{s}}{\rho_{f}}\right)}\right)}{{\left(1-\phi +\frac{\phi \rho_{s}}{\rho_{f}}\right)}^{-1}}$$

(22) $$W_{s}=-\frac{W_{0}}{2\Omega_{1}h},\;Ec=\frac{{\left(\Omega_{1}r\right)}^{2}}{c_{p}\left(T_{1}-T_{2}\right)},\;Rd=\frac{16\sigma^{*}T_{\infty }^{3}}{3k_{f}k^{*}},\;\Omega =\frac{\Omega_{1}}{\Omega_{2}},\;Re=\frac{h^{2}\Omega_{1}}{\nu_{f}},\;A_{2}=\frac{a_{2}}{\Omega_{2}},\;Pr=\frac{\nu_{f}{\left(\rho c_{p}\right)}_{f}}{k_{f}},\;A_{1}=\frac{a_{1}}{\Omega_{1}}.\;$$

### 3.5. Significant Quantities from for Engineers

The wall shear stresses attained significant importance in various technological and engineering areas. For the model of nanofluid under consideration, the wall shear stresses in dimensional form are expressed in the following mathematical formulas:

(23) $$\tau_{zr}^{*}=\mu_{nf}^{*}\frac{\partial u^{*}}{\partial z}{↓}_{z=0}=\frac{\mu_{f}^{*}\Omega r_{}}{h\left(1-34.87{\left(\frac{d_{particle}}{d_{fluid}}\right)}^{-0.3}\phi^{1.03}\right)}F^{\prime \prime }\left(\eta \right){↓}_{\eta =0}$$

(24) $$\tau_{z\theta }^{*}=\mu_{nf}^{*}\frac{\partial v^{*}}{\partial z}{↓}_{z=0}=\frac{\mu_{f}^{*}\Omega r_{}}{h\left(1-34.87{\left(\frac{d_{particle}}{d_{fluid}}\right)}^{-0.3}\phi^{1.03}\right)}\;G^{\prime }\left(\eta \right){↓}_{\eta =0}$$

The total shear stresses at the upper and lower are given by the following mathematical relation:

(25) $$\tau_{w}^{*}={\left(\tau_{zr}^{*}{}^{2}+\tau_{z\theta }^{*}{}^{2}\right)}^{1/2}$$

By plugging the similarity transformations and after simplification, the following relations for the shear stresses at the upper and lower disks are obtained:

(26) $$C_{F_{0}lower}=\frac{\tau_{w{↓}_{z=0}}^{*}}{{\left(r\Omega_{1}\;\right)}^{2}\rho_{f}^{*}}=\frac{\sqrt{F^{\prime \prime }{\left(\eta \right)}^{2}+G^{\prime }{\left(\eta \right)}^{2}}}{\left(1-34.87{\left(\frac{d_{particle}}{d_{fluid}}\right)}^{-0.3}\phi^{1.03}\right)Re_{r}}\;\mathrm{at}\;\eta =0$$

(27) $$C_{F_{1}upper}=\frac{\tau_{w{↓}_{z=h}}^{*}}{{\left(r\Omega_{1}\;\right)}^{2}\rho_{f}^{*}}=\frac{\sqrt{F^{\prime \prime }{\left(\eta \right)}^{2}+G^{\prime }{\left(\eta \right)}^{2}}}{\left(1-34.87{\left(\frac{d_{particle}}{d_{fluid}}\right)}^{-0.3}\phi^{1.03}\right)Re_{r}}\;\mathrm{at}\;\eta =1$$

### 3.6. Bejan Effects Modelling

The mathematical relation for entropy generation is described as follows:

(28) $$S_{G}^{*}=\frac{k_{f}^{*}\;\left(\frac{k_{nf}^{*}}{k_{f}^{*}}{\left(\frac{\partial T^{*}}{\partial z}\right)}^{2}+\frac{16\sigma^{*}T_{\infty }^{3}}{3k^{*}k_{f}^{*}}{\left(\frac{\partial T^{*}}{\partial z}\right)}^{2}\right)}{T_{f}^{*2}}++\frac{\mu_{nf}^{*}\Phi^{*}}{T_{f}^{*}}$$

By plugging the viscous dissipative term in Equation (28), the following relation is attained:

(29) $$S_{G}^{*}=\frac{k_{f}^{*}\;\left(\frac{k_{nf}^{*}}{k_{f}^{*}}{\left(\frac{\partial T^{*}}{\partial z}\right)}^{2}+\frac{16\sigma^{*}T_{\infty }^{3}}{3k^{*}k_{f}^{*}}{\left(\frac{\partial T^{*}}{\partial z}\right)}^{2}\right)}{T_{f}^{*2}}+\frac{\mu_{nf}^{*}\left(2\left({\left(\frac{\partial u^{*}}{\partial z}\right)}^{2}+{\left(\frac{u^{*}}{r}\right)}^{2}+{\left(\frac{\partial w^{*}}{\partial z}\right)}^{2}\right)+{\left(\frac{\partial v^{*}}{\partial z}\right)}^{2}+{\left(\frac{\partial w^{*}}{\partial r}+\frac{\partial u^{*}}{\partial z}\right)}^{2}+{\left(r\frac{\partial }{\partial r}\left(\frac{v^{*}}{r}\right)\right)}^{2}\right)}{T_{f}^{*}}$$

After this, plugging the similarity transformations and suitable partial differentiation, the following self-similar form is obtained:

(30) $$N_{G}^{*}=\left(\frac{k_{nf}^{*}}{k_{f}^{*}}+Rd\right)\alpha_{1}\beta^{\prime 2}+\frac{Br\left(12F^{\prime 2}+G^{\prime 2}A+F^{\prime \prime 2}A\right)}{\left(1-34.87{\left(\frac{d_{particle}}{d_{fluid}}\right)}^{-0.3}\phi^{1.03}\right)Re}$$

Moreover, the mathematical expression for the Bejan number is as follows:

(31) $$Be=\frac{\left(\frac{k_{nf}^{*}}{k_{f}^{*}}+Rd\right)\alpha_{1}\beta^{\prime 2}}{\left(\frac{k_{nf}^{*}}{k_{f}^{*}}+Rd\right)\alpha_{1}\beta^{\prime 2}+\frac{Br\left(12F^{\prime 2}+G^{\prime 2}A+F^{\prime \prime 2}A\right)}{\left(1-34.87{\left(\frac{d_{particle}}{d_{fluid}}\right)}^{-0.3}\phi^{1.03}\right)Re}}$$

The dimensionless physical parameters appear in Equations (31) and (32), as follows:

(32) $$A={\left(\frac{r}{h}\right)}^{2},\;Br=\frac{\mu_{f}^{*}{\left(h\Omega_{1}\right)}^{2}}{\Delta T^{*}k_{f}^{*}},\;N_{G}^{*}=\frac{\nu_{f}^{*}S_{G}^{*}T_{f}^{*}}{\Omega_{1}\Delta T^{*}k_{f}^{*}},\;\alpha_{1}=\frac{\Delta T^{*}}{T_{f}^{*}},\;where\;\Delta T^{*}=T_{1}^{*}-T_{2}^{*}$$

### 3.7. Mathematical Analysis

The nanofluid model under consideration, comprising the influences of freezing temperature, molecular and nanomaterials diameters, is very tedious, highly nonlinear and coupled system of ordinary differential equations. It is not possible to find the exact solutions for such a nanofluid model. Thus, the numerical techniques are best to tackle the model of such nature. Therefore, Runge Kutta (RK) numerical technique with coupling shooting technique [24,25] was adopted for mathematical treatment of the particular nanofluid model over the domain of interest. The initial step for the implementation of the aforementioned technique is transformations that transform the model under consideration into a system of first-order ordinary differential equations, and then solves them for the physical results. These transformations are taken in the following pattern, for our nanofluid model:

(33) $$\begin{array}{c} b_{1}^{*}=F,\;b_{2}^{*}=F^{\prime },\;b_{3}^{*}=F^{\prime \prime },\;b_{4}^{*}=F^{\prime \prime \prime }\\ b_{5}^{*}=G,\;b_{6}^{*}=G^{\prime }\\ b_{7}^{*}=\beta ,\;b_{8}^{*}=\beta^{\prime } \end{array}\}$$

## 4. Outcomes

The analysis of radiative colloidal fluid Al_2_O_3_-H_2_O by incorporating the influences of nanomaterials diameter, viscous dissipation and entropy generation for thermal enhancement is of much interest and has gained the popularity among the engineer and researcher communities. Specifically, the analysis of entropy generation in colloidal fluids is significant from the point of view of heat engines, air conditioners and refrigeration. Therefore, the study is presented in light of the aforementioned applications. From the presented work, the following is detected:

- The axial velocity G(η) of Al_2_O_3_-H_2_O upturns due to the stretching of lower end over 0≤η≤1.
- Thermal transport enhances for B_r_ and is detected that more dissipative Al_2_O_3_-H_2_O colloidal fluid is better for industrial and engineering applications where huge amount of heat transfer is required to acquire the process of production.
- The entropy generation due to an irreversible process such as fluid flow and heat transfer enhances in Al_2_O_3_-H_2_O for multiple values of α.
- The effects on the colloidal flow of Al_2_O_3_-H_2_O enhances for strong thermal radiations phenomena.
- The effective characteristics of the colloidal fluid enhances for fraction factor ϕ of the nanomaterial, which significantly enhances the thermal transportation in Al_2_O_3_-H_2_O.
- Due to stronger fraction factor, ϕ, more colloidal fluid Al_2_O_3_-H_2_O particles drag at the surface are detected.

Future Insights: The modern world is moving toward the applications of nanotechnology by considering numerous physical phenomenon, almost from every aspect of life. Therefore, the analysis of Lorentz forces, resistive heating and double stratification that are extensively applied in the production industries are the topics of interest in the study of nanofluids these days.

## Figures and Tables

**Table 1:** C_F0_ and C_F1_ for multiple parameters.

$A_{1}$	ϕ	$A_{2}$	$A_{3}$	Re	$C_{F0}$	$C_{F1}$
0.1	0.1	0.1	0.1	2.0	1.83064119961189	2.35269863209794
0.3					2.92947464900369	2.74689705930503
0.5					4.06331585276896	3.14645174737726
0.1	0.13				1.82035367595433	2.37595226538747
	0.16				1.81047026581729	2.39925198398080
	0.19				1.80099131033662	2.42259739262078
	0.10	0.3			2.01427466585367	3.30675284677154
		0.5			2.21780156926006	4.29179421504348
		0.7			2.44566929988645	5.31193688240473
		0.1	0.3		1.41884526339252	2.08906895306680
			0.5		1.17855950472776	1.84739804212192
			0.7		1.11249451452106	1.62705943248726
			0.1	3.0	1.76281621068053	2.52985585298949
				4.0	1.71666966059284	2.71621873855048
				5.0	1.69316443574177	2.91363132459533

**Table 2:** Thermophysical characteristics of the nanoparticles and host liquid at temperature T = 310 K [22].

Properties	$d_{p}\left(nm\right)$	$\rho \left(kg/m^{3}\right)$	β (1/k)	$c_{p}\left(J/Kg\;K\right)$	$\mu_{f}\;\left(kg/ms\right)$	k(W/mk)	σ(S/m)
$H_{2}O$	0.385	993	$36.2\times 10^{5}$	4178	$695\times 10^{6}$	0.628	0.005
Al_2_O_3_	33	3970	$0.85\times 10^{5}$	765	---------	40	$0.05\times 10^{6}$

## Nomenclature

$\rho_{nf}^{*}$	effective density
$\mu_{nf}^{*}$	effective dynamic viscosity
$k_{nf}^{*}$	effective thermal conductivity
${\left(\rho c_{p}\right)}_{nf}$	effective heat capacity
nf	stands for nanofluid
$\rho_{p}$	density of nanomaterial Al_2_O_3_-H_2_O
$\rho_{f}$	density of host liquid
ϕ	fraction factor of the nanomaterial
$k_{p}$	thermal conductivity of Al_2_O_3_
$k_{f}$	thermal conductivity of the host liquid
${\left(\rho c_{p}\right)}_{p}$	heat capacity of Al_2_O_3_
${\left(\rho c_{p}\right)}_{f}$	heat capacity of host liquid
$T_{1}$	temperature at lower disk
$T_{2}$	temperature at upper disk
$\Phi^{*}$	viscous dissipation
p	pressure
$d_{p}$	diameter of nanomaterial
$k_{b}$	Stefan Boltzmann coefficient
η	similarity variable
F(η)	axial velocity
$F^{\prime }\left(\eta \right)$	radial velocity
β(η)	dimensionless temperature field
Ec	Eckert number
Re	Reynolds number
Rd	Radiation parameter
Pr	Prandtl number
Be(η)	Bejan number
$N_{G}\left(\eta \right)$	Entropy generation
